# Content shared on social media for national cancer survivors day 2018

**DOI:** 10.1371/journal.pone.0226194

**Published:** 2020-01-15

**Authors:** Roy Cherian, Gem Le, James Whall, Scarlett Gomez, Urmimala Sarkar

**Affiliations:** 1 Department of Medicine, Division of General Internal Medicine University of California, San Francisco, Zuckerberg San Francisco General Hospital, San Francisco, California, United States of America; 2 California Northstate University College of Medicine, Elk Grove, California, United States of America; 3 Department of Epidemiology and Biostatistics, University of California, San Francisco, San Francisco, California, United States of America; University of Sussex, UNITED KINGDOM

## Abstract

**Background:**

Studies estimate that the number of cancer survivors will double by 2050 due to improvements in diagnostic accuracy and treatment efficacy. Despite the growing population of cancer survivors, there is a paucity of research regarding how these individuals experience the transition from active treatment to long-term surveillance. While research has explored this transition from more organized venues, such as support groups for cancer survivors, this paper explores the discourses surrounding cancer survivorship on social media, paying particular attention to how individuals who identify as cancer survivors represent their experience.

**Methods:**

We identified social media posts relating to cancer survivorship on Twitter and Instagram in early June 2018, in order to coincide with National Cancer Survivorship Day on June 3, 2018. We used nine pre-selected hashtags to identify content. For each hashtag, we manually collected the 150 most recent posts from Twitter and the 100 most recent plus the top 9 posts from Instagram. Our preliminary sample included 1172 posts; after eliminating posts from one hashtag due to irrelevance, we were left with 1063 posts. We randomly sampled 200 of these to create a subset for analysis; after review for irrelevant posts, 193 posts remained for analysis (118 from Instagram and 75 from Twitter). We utilized a grounded theory approach to analyze the posts, first open-coding a subset to develop a codebook, then applying the codebook to the rest of the sample and finally memo writing to develop themes.

**Results:**

Overall, there is substantial difference in the tone and thematic content between Instagram and Twitter posts, Instagram takes on a more narrative form that represents journeys through cancer treatment and subsequent survivorship, whereas Twitter is more factual, leaning towards advocacy, awareness and fundraising. In terms of content type, 120 posts (62%) of the sample were images, of which 42 (35%) were images of the individual posting and 28 (23%) were images of patients posted by family or friends. Of the remaining images, 14 (12%) were of support groups and 7 (6%) were of family or friends. We identified four salient themes through analysis of the social media posts from Twitter and Instagram: social support, celebrating milestones and honoring survivors, expressing identity, and renewal vs. rebirth.

**Discussion:**

We observed a marked relationship between physical appearance, functional status and survivorship. Additionally, our findings suggest the importance of social support for cancer patients and survivors as well as the role social media can pay in identity formation.

**Conclusion:**

Our findings suggest that individuals who identify as survivors on social media define their identity fluidly, incorporating elements of physical, emotional and psychological health as well as autonomy.

## Introduction

In 2016 there were an estimated 15.5 million cancer survivors and that number is expected to double by 2050 due to improvements in screening and treatment.[1, 2]

As a point of departure, a keystone report on cancer survivorship by the Institute of Medicine outlined a variety of issues facing cancer survivors and recommendations on how to address those issues.[1] Cancer survivors are complex patients with a variety of long-term risks and comorbidities including cardiovascular disease and damage, decreased bone health, hematologic dysfunction, changes in appearance, distress, changes in sexuality and fertility, treatment side effects, and necessary lifestyle changes.[3,4] The multitude of changes that accompany cancer treatment often precipitates in psychosocial distress as well,[5] including but not limited to fear of recurrence.[6]

As the number of survivors increases, the medical field must prepare to manage the complex issue of survivorship. However, research has lagged in exploring, let alone addressing, the complex care needs of this growing population. In part, this gap can be attributed to the focus on treatment and prevention in cancer research, which does not easily accommodate the ambiguous sick role of the cancer survivor,[7,8] who has completed treatment and for whom prevention is framed in terms of recurrence. In particular, the psychosocial effects of cancer survivors [1] have received considerably less attention than clinical outcomes. Given the biopsychosocial nature of illness and recovery,[9] this gap demands redress especially as improvements to screening and treatment continues to improve outcomes and survival.

Lacking a definite space within the health system, cancer survivors often form peer support groups, both virtual and in person.[10–12] Previous studies have shown how these venues can improve coping and quality of life [13–15]; with the advent of social media, the sites both impromptu and organized peer support and intervention are growing. In 2019, 37% of adults use Instagram and 22% used Twitter; however, the bulk of this is among adults between the ages of 18 and 49, which is below the average age of cancer diagnosis in the United States.[16,17] Nevertheless, these platforms provide the opportunity and space to candidly share illness narratives related to cancer and are worth exploring. The increasing use of social media and the role it plays in identity formation,[18,19] therefore, offers a potential avenue to explore the lives of cancer survivors as they are being and becoming. In particular, social media may provide a window into the non-clinical aspects of cancer survivorship. However, to date, cancer research on social media has continued the trend of focusing largely on screening and prevention,[20] leaving survivorship to the wayside.

To explore these contemporary venues for representation, communication and peer support, this paper aims to analyze social media posts related to cancer survivorship broadly. More specifically, while there have been studies related to online peer support groups,[21,22] we are interested in how individuals who identify as cancer survivors derive meaning from their experiences of treatment. We hypothesize that social media provides a venue for these individuals to represent meaning organically, or external to any organized virtual space or community that may structure or condition representations within its own discursive field, thereby closing off other forms of meaning and representation. For example, a forum focused on breast cancer and fitness potentially proscribes narratives that speak to the extreme difficulty of maintaining an active lifestyle during or after treatment. Moreover, although studies have described narratives of cancer survivorship on YouTube or organizational accounts on Twitter,[23,24] personal accounts on impromptu spaces like Twitter or Instagram remain relatively unexplored. Unlike YouTube, which requires a certain level of planning and skills in video production and editing, platforms such as Instagram and Twitter give researchers a window into how individuals who identify as cancer survivors construct meaning from their experiences of treatment though the words, images, and videos on platforms that promote usability and engagement. In aggregate, these posts may create a window to better understand the meaning of survivorship for cancer patients as a whole.[25–27] However, just because social media platforms aren’t mediated in the same way as traditional venues (e.g. support groups) or research activities (e.g. focus groups) does not mean that users are not conditioned to represent themselves in a particular way based on perceptions of what is normative or expected. [28]

## Methods

### Data collection

We identified social media posts relating to National Cancer Survivors Day and National Cancer Survivors Month. Previous studies on prostate and testicular cancers suggest that using events (e.g. Movember) to drive sampling could be a fruitful approach for identifying narratives and representations of interest.[29,30] Using a previously published method of hashtag identification,[31] we identified 9 relevant hashtags, or words or phrases preceded by the “#” symbol that allow social media platforms to aggregate posts,[32] from Twitter and Instagram (#nationalcancersurviorsday, #ncsd2018, #survivorshipmonth, #endcancer, #cancersurvivorsday, #cancersurvivor, #cancercommunity, #bethebossovercancer, #celebratelife). Two researchers (GL and RC) identified these hashtags by first finding which hashtags were trending on June 3, 2018 (e.g. #nationalcancersurvivorsday, #ncsd2018 and #cancersurvivorsday) and then using these to identify others that commonly co-occurred with these. Before including these secondary hashtags, we ensured that they consistently identified posts that described survivorship narratives and experiences.

We did not use the streaming application program interface (API) for Twitter or Instagram but instead used screenshots, supplemented with transcription for videos, to manually gather posts under each hashtag from the platforms. Data collection was completed in a single day, June 11, 2018 and included posts shared between May 27, 2018 and June 10, 2018. Within this sampling window, we took screenshots of 150 posts from Twitter and 100 posts on Instagram for each hashtag, starting with the most recent. Some hashtags were not represented throughout the sampling window, but rather only peaked on June 3, 2018 (NCSD). We also included the 9 most popular Instagram posts under each hashtag insofar as they were shared within our sampling window. We chose these numbers (i.e. 150 from Twitter and 109 from Instagram) at random and not all hashtags had 150 Twitter or 109 Instagram posts associated given variable prevalence throughout the sampling window. Additionally, hashtags were not mutually exclusive, leading us to exclude duplicates. In total, we collected 437 posts from Twitter and 735 posts from Instagram. Our initial sample prior to review included a total 1172 posts (735 Instagram; 437 Twitter). All data collection complied with the terms of service for Twitter and Instagram.

**Fig 1:**
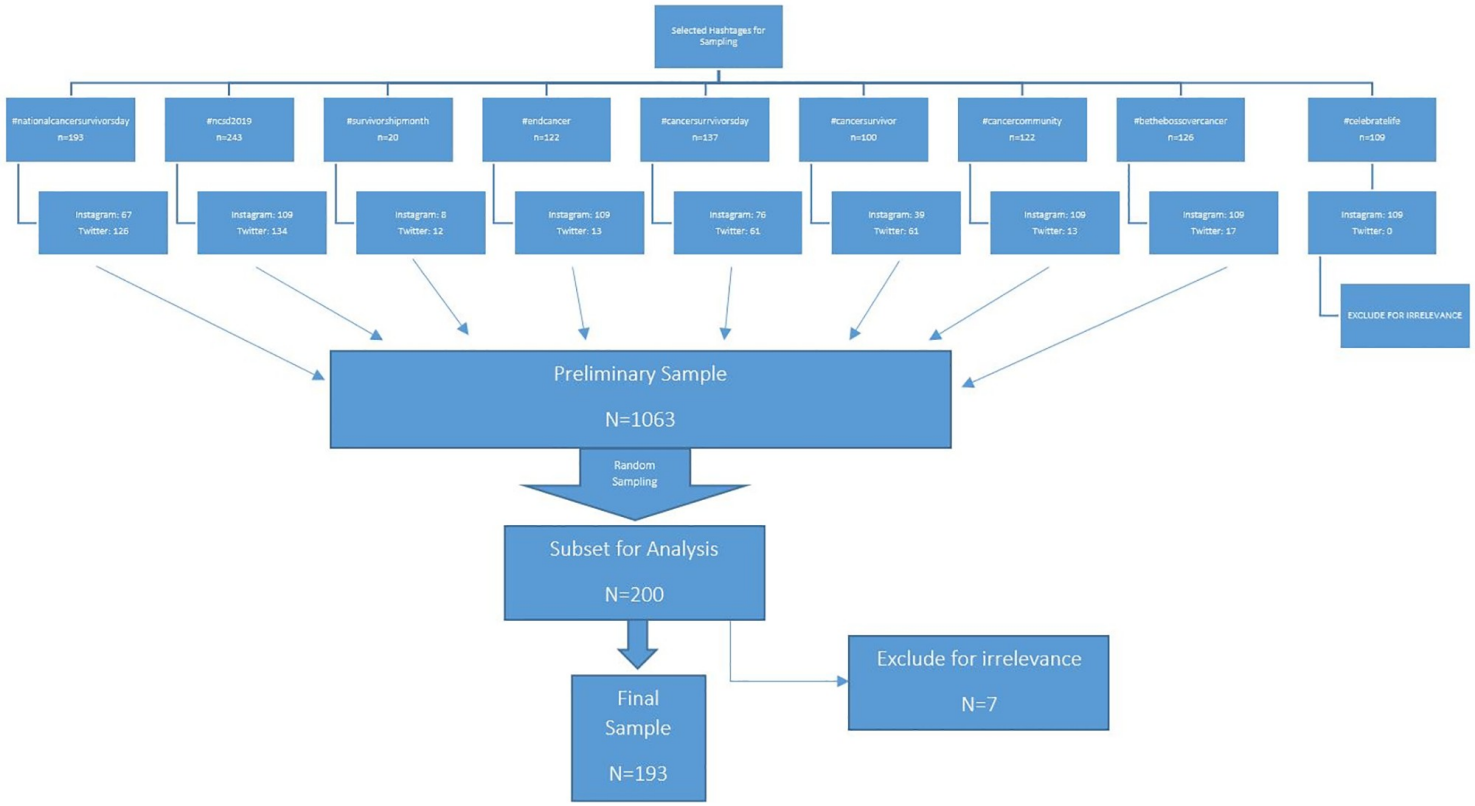
Sampling strategy.

After review of the initial sample, we excluded posts with the hashtag #celebratelife for a lack of specificity in targeting cancer-related posts. This yielded a final dataset of 1063 posts, 437 from Twitter and 626 from Instagram. To create our subset for analysis, we randomly selected 200 posts from this dataset by assigning a random number to each post, sorting them from smallest to largest and then selecting the first 20% of posts within each hashtag, aggregating Twitter and Instagram. After excluding non-cancer related posts, our final sample was 193 (118 Instagram; 75 Twitter). Please see Fig 1 for details regarding sampling strategy.

As this study used de-identified, publically available social media data, the UCSF IRB classified our proposal as exempt from review and waived the requirement for informed consent. While re-identification is a challenge presented by social media research, contacting all posters for consent individually is not feasible. Moreover, conducting a closed study that explicitly aims to solicit posts related to survivorship on social media from consented individuals would not allow us to get at the organic conversations surrounding survivorship presented in this paper. Therefore, de-identification is the best we are able to do to promote patient confidentiality and privacy.

### Analysis

We applied a grounded theory methodology and a phenomenological approach to analyze a randomly sampled subset of 193 posts. More specifically, we used the methods of open-coding, memo writing and grouping to understand how the social media phenomenon of National Cancer Survivor’s Day structures experiences, and subsequent narratives, of undergoing cancer treatment. Therefore, the purpose was not to objectively characterize survivorship but rather to ascertain the ways NCSD on social media structures meaning of survivorship. To standardize coding methods, we included short descriptions and summaries for videos, which we coded for general content (e.g. exercise). RC and GL both open-coded the first 50 and then compared annotations to determine the data was being interpreted in a similar way. Once this was established, RC and GL went through the preliminary codes to develop a codebook (S1 Appendix) to apply to the remaining 143 posts, which researchers divided evenly and reviewed independently. After RC and GL coded all of the posts, they met once more to go over code application. Subsequently, RC and GL wrote memos independently to begin drawing out themes from the data by grouping codes into thematic categories. Lastly, RC and GL met to compare and discuss the memos, consolidate categories and finalize themes. Drawing on a traditional grounded theory approach,[33,34] it should be noted that each theme is made up of one or more categories, which themselves are made up of multiple codes. Therefore, all of the themes presented represent the responses of multiple individuals, groups and/or organizations.

## Results

The greatest percentage posts came from patients who identify as survivors themselves (93; 48%) and spoke about cancer survivorship generically (131; 68%). The remaining posts referenced breast (31; 16%), sarcomas (6; 3%), prostate (6; 3%), ovarian (5; 3%), colorectal (3; 2%), lung (2; 1%), testicular (2; 1%), lymphoma (2: 1%), cervical (2; 1%), kidney (1; 0.5%), esophageal (1; 0.5%) and eye (1; 0.5%) cancers. Table 1 describes the types of posts in aggregate and across platforms. While all posts included some sort of textual content, we classified posts by their dominant features. In terms of content type, 120 posts (62%) of the total sample were images, of which 42 (34%) were images of the individual posting and 28 (23%) were images of patients posted by family or friends. Of the remaining images, images of support groups (14; 12%) and family or friends (7; 6%) were the most prominent, while there were a number of smaller categories. Please see Table 2 for full description; for definitions please refer to S2 Appendix. We determined categories through open coding and subsequent grouping of open-codes into categories. Therefore, posts classified as text contained only text whereas posts classified as “image” or “video” contained text as a caption to the multimedia content.

**Table 1:** Content type.

Content Type	Total # (%)	Instagram # (%)	Twitter # (%)
Image	120 (62%)	87 (74%)	33 (44%)
Text	62 (32%)	20 (17%)	42 (56%)
Video	11 (6%)	11 (9%)	0 (0%)
Total	193 (100%)	118 (100%)	75(100%)

**Table 2:** Overall image content.

Photo Subject	Total # (%)	Instagram # (%)	Twitter # (%)
Selfie*	**42 (34%)**	**40 (46%)**	2 (6%)
Patient	26 (21%)	18 (21%)	**8 (24%)**
Support Group	14 (11%)	6 (7%)	**8 (24%)**
Promotion/Advertising	10 (8%)	5 (6%)	5 (15%)
Family/Friends	7 (6%)	4 (5%)	3 (9%)
Nature	5 (4%)	1 (1%)	4 (12%)
Nutrition	4 (3%)	4 (5%)	0 (0%)
Doctor	3 (2%)	2 (2%)	1 (3%)
Fundraising	3 (2%)	2 (2%)	1 (3%)
Fitness	2 (2%)	2 (2%)	0 (0%)
Information	2 (2%)	1 (1%)	1 (3%)
Other	2 (0%)	2 (2%)	0 (0%)
TOTAL	120	87 (100%)	33 (100%)

*we classified posts as “selfies” if the patient took the picture(s) included in the post him/her/themselves, whereas we classified posts as “patient” if friends or family took the photo of the patient.

Overall, we observed a stark difference in the tone and thematic content in Twitter postings compared to the Instagram postings. The majority of Instagram posts (96/118; 81%) took on a more narrative form that represents journeys through cancer treatment and subsequent survivorship through the use of personal anecdotes and selfies, whereas the majority of Twitter posts (59/75; 79%) were more factual, leaning towards advocacy, awareness and fundraising, often utilizing professional images and logos. Given this difference and our intention to explore the meanings of survivorship for cancer patients, the following analysis focuses primarily on the richer, more personal, data from Instagram.

### Social support

One of the most salient themes to emerge from the data is the role of social support both during and after treatment. We coded and categorized posts that described family, friends and other cancer patients as sources of strength and inspiration that helped carry survivors through treatment as representing social support:

*I was strong most days but I had great people in my corner for those days I wasn’t*.

(Instagram)

Posts described the critical role of social support on the entire trajectory from diagnosis along the treatment pathway to the recovery and survivorship stages.

*Upon finding out the news I was terrified and didn’t know what to make of it. Even if I had the power to change myself having cancer, I wouldn’t. It has taught me many lessons at a very young age that I may have never learned. This experience brought me closer to everyone around me. Everyday I am learning to love myself and my life. I have met amazing people through this experience such as my doctors+ nurses and cancer patients + survivors + ostomy patients throughout the entire world. Being able to connect with cancer patients, survivors, and anyone living with an ostomy brings me so much joy. I wish to help give them hope to live a long and healthy live and lift them up when they feel weak*.

(Instagram)

Peer support in particular is among the most impactful forms of social support for cancer patients and survivors, whose family and friends are often unable to understand what the patient may be going through:

*#Breastcancer Patient Advocacy Tip #3*. *Find another #cancersurvivor and let them love you through your journey*. *Make connections and share your feelings with someone who understands*!

(Twitter)

Despite the importance of peer support, posters also described the importance of supportive and compassionate caregivers, who by stepping outside their clinical duties in simple ways inspired hope and resilience in survivors:

*Without this experience I wouldn’t have met one of the closest people to me*, *my nurse [Name]*. *I am grateful for how much she cares for me*, *rubbing my back the days I didn’t feel well*, *to the days she visited me in the hospital*, *always being there to talk to on a personal level*, *to giving me hope for a beautiful life*, *as well as walking with me after I had my surgery*, *and everything else she does for me*. *I will never forget the day I was laying in the hospital bed with the lights out and blinds covering the windows*, *when [Name] reminded me that I had a chance that some people didn’t have*. *Reminding me that I have the rest of my life ahead of me and that I was just hitting a bump in the road*. *I have never stopped thinking about this day since and it inspires me everyday to live for those who have passed away and cancer patients who are fighting everyday for their lives*.

(Instagram)

While the majority of non-organizational posts came from patients and survivors themselves, on occasion family or friends would post describing how they have been inspired by the struggles of a loved on going through cancer treatment:

*At the age of 13 when girls were stressing about slumber parties*, *ballet classes and school*, *you were stressing about chemotherapy*, *nausea*, *and hair loss*. *We learned what osteosarcoma was*, *and in the process also learned what strength and faith looked like*. *You were a fighter then and continue to be an inspiration to us all*. *Today is National Cancer Survivors Day and I celebrate you my beautiful [NAME]*. *May you inspire all those fighting against this horrible disease!*

(Instagram)

Overall, cancer patients describe how social support from family, friends, caregivers and other patients as critical to their ability to endure treatment and the uncertainty that accompanies it.

### Celebrating milestones and honoring survivors

As expected based on the key words we used for National Cancer Survivors’ Day (NCSD), we saw that “celebration” is a recurring theme across the sample of Instagram and Twitter posts. In doing so, some posters problematized the concept of survivorship as too narrow. Specifically, posts emphasized that life-post cancer was to be lived to the fullest rather than simply endured as “surviving” implies:

*Life after cancer is more than just surviving*. *It’s about living*. *It’s celebrating milestones*.

(Instagram)

Moreover, posters implied that survivorship should set aside the anxiety of recurrence and counting the years but rather focus on imbuing each moment, however brief, with value and significance:

*Life after cancer is about more than just counting years*. *It’s about making those years count*.

(Instagram)

In addition to cancer remission, treatment completion and initiation were described milestones that are cause for celebration, with survivorship sometimes referenced aspirationally (e.g. “just finished up chemo day one #wearesurvivors”). Often the focus on celebration was paralleled with the more somber theme of honor and remembrance, wherein celebrating the triumph of survivors over cancer was juxtaposed with the commemoration of those who had passed.

*Today is #NationalCancerSurvivorsDay*. *Today we celebrate the people who have overcome #cancer*, *we inspire those who are currently facing it*, *and we honor those who have passed*. *Every single experience with cancer that is shared here is a source of strength for someone else*. *If you have a before and after picture to share today*, *use the hashtag #LifeAfterDiagnosis and the #NCDS and tag us @ihadcancer for a chance to be featured on our page!*

(Instagram)

### Expressing and forming identity

Our findings suggest that the experiences of cancer diagnosis, treatment and subsequent survivorship represented on social media are salient in the identity formation of cancer survivors,[18] who describe these as changing their relationship with themselves and loved ones as well as their orientation to life and death more broadly. Among the most common identities adopted by cancer patients and survivors is that of a “warrior” or “fighter”—a common theme in biomedical paradigms of healing.[35] For these individuals, the struggle against cancer becomes internalized as a defining part of their lived experience and identity:

*Today we recognize all those who bravely take on a battle they never asked for*. *Share your pride*, *loud and proud!*

(Instagram)

Couldn’t be happier to be here than I am today. 21 months ago I was diagnosed with #ovariancancer and had surgery and chemotherapy. During the course of chemo I contracted sepsis. There could have been a totally different outcome to my story, but today I am in remission, thriving and feeling great

(Instagram)

In contrast to military metaphors, patients and survivors also described cancer as a journey:

*Today is #NationalCancerSurvivorsDay*. *Celebrating 4 years as a survivor*. *Thankfuly for my journey and the strength I’ve found along the way*. *Thankful for all of the family and friends who have walked this journey with me*. *God is so faithful and I am blessed beyond words to live this beautiful life of mine*.

(Instagram)

Describing cancer as a journey is an emergent trend,[36] which has been argued to minimize feelings of guilt or failure that are implicitly felt by those who conceptualized themselves as “fighters” or “warriors” if treatment is ineffective. By displacing agency away from the patient towards external forces that shape the journey, patients place trust in others including family, friends, caregivers and their religious faith.

To emphasize the transformative experience of cancer diagnosis and treatment, it was also common to see posts showing change over time or contrasting treatment and recovery alongside notions of gratitude and the fragility of life:

*(@)Repost from @XXXXX_—Today is National cancer survivor day*. *These pictures are 5 years apart*, *I have come a long way*. *Cancer forever changes your life*, *and I am GRATEFUL for everything single day since*. *Life isn’t always easy*, *and tomorrow isn’t promised*. *So love your tribe hard*, *and find joy in the small things ❤💕💪*

(Instagram)

Posts often merged physical transformations resulting from receiving a cancer diagnosis and undergoing treatment with the aforementioned theme of celebration and commemoration:

*(@)Repost from @XXXXX Today is National Cancer Survivors Day*. *Life after cancer is about more than just counting years*. *It’s about making those years count*. *Life can change in a blink of an eye*. *Top Left photo was October 2015 when I woke up in the ICU with chemo dripping into my body and facing reality of stage 4 cancer*. *Bottom left photo was January 2016 right after coming out of chemo at my lowest and weakest point in life*. *Not knowing where my life was going or if I would make it to see another day*. *And then there is today*. *Grateful to be alive*. *It is still a struggle everyday mentally*, *emotionally and physically*. *Not a day goes by I don’t think of the ones we loss in this beautiful life to cancer*. *My heart and soul ache for those we’ve lost and the ones battling*. *Cancer sucks*. *As I continue to live each day I try to find the Peace in every day*. *Most of all I tell Cancer to PEACE Out for all cancer awareness*. *✌💚*

(Instagram)

However, despite the role of cancer diagnosis and treatment plays in identity formation, it is clear that “cancer survivor” is only one lens through which posters defined themselves. Instead, we gleaned that cancer is a transformative experience that cannot be narrowly defined:

*I don’t usually define myself as a cancer survivor*. *I don’t keep up on cancer events or participate in fund raising or wear pink pins*. *Cancer is part of my past*. *God used it to change who I am*. *I am not the same young woman I was when I got my first positive biopsy results*. *I’m better than who I was*. *For that I will be forever grateful*.

(Instagram)

While not identifying as a cancer survivor or engaging in commemorative or fundraising activities, this poster describes the role cancer played in transforming her into a different and better person. This post, as well as many others, problematize the concept of survivorship as too narrow, and perhaps too focused on the past.

### Rebirth vs. renewal

While identity formation can describe the function of these posts broadly, more specifically individuals described their post-treatment identities and experience in terms of rebirth and/or renewal. Many representations of survivorship embrace the changes brought about by cancer treatment—both temporary and permanent—such as hair loss and scarring. In addition to having gained strength and perspective, cancer survivors describe emerging from treatment renewed or reborn.

*There is a misconception that when a patient is “cancer free*,*” they can move on with their life*. *But for kids like 2014 Ambassador _____*, *the shadow of cancer is always there*. *“It’s been difficult at times*, *but I’m grateful to be alive*. *These challenges I’ve experienced have only helped me to become a stronger person*.*” To kick off National Cancer Survivors Month*, *take a peek into _____s life as a childhood cancer survivor*:

(Instagram)

In particular, we identified posts where scarring and hair loss were accompanied by a smile or otherwise bold face. In addition, we observed multiple instances of breast cancer survivors sharing nude, post-surgical images, often accompanied by statements of self-affirmation, gratitude and acceptance.

*So today (June 3rd) is National Cancer Survivor day* … *Each day is another day of survival*. *I am at a point where I appreciate my body* … *I am not ashamed to flaunt my not so perfect post-#mastectomy boobs*. *Sometimes it’s not about what happened* … *or how I look*.. *for me*.. *it’s about being here to tell my story*. *I used to think days like today would never come*. *I am so grateful for this trip of solitude*. *I needed it* … *I haven’t relaxed YEARS*. *Get out of that comfort zone guys*. *Nothing great happens without risk❤*

(Instagram)

In additional to challenging conventional notions of beauty, posters thought it important to emphasize the difficulties of undergoing treatment that are often glossed over and willfully forgotten:

*Standing at 5’10 I weighed 116 lbs*. *My normal weight is 137*. *I had already lost my hair*, *eyebrows and lashes due to chemo and it was just starting to grow back*. *I had to break into my mother’s photos in order to even find these pics bc I hated the way that I looked and pictures were just a reminder to me*.*I felt that it was important to post these for myself and for the people who didn’t get to see me during this stage and also for those who don’t know me*. *It occurred to me that each photo of me that I found* …*I always had on a smile and that’s how I got through everything I’ve survived*.

(Instagram)

Despite these re-imaginings of beauty, the most popular posts represented the line between treatment and survivorship as a return to previous appearance and functional status, with many posts emphasizing “#thisisme”.

## Discussion

To put our findings in the context of literature on social media and cancer to date, aligned with previous studies, our analysis of posts from National Cancer Survivors Day demonstrates how cancer awareness months promote awareness by the sharing of illness narratives and treatment trajectories on social media.[20,26,27,29].

In these representations, cancer survivors engage consumers of social media with narrative elements such as dramatic tension and the climactic loss of agency.[23] By juxtaposing treatment and post-treatment functional status and appearance, survivors respectively associate the general loss of autonomy with a renewed capacity for representation. In other words, these narratives recalling early stages of treatment from the perspective of survivorship link shifts in agency and autonomy with milestones in the treatment pathway.

Specifically, we observed the salience of social support both during and after treatment, a theme that features prominently in the literature.[37–42] Given the associations between social isolation and cancer mortality,[43] the importance of social support for cancer patients cannot be overstated. One could argue that social media provides a venue for one of the most overlooked aspects of cancer care: social support. For example, individuals who identify as cancer survivors may experience comments, likes and shares on their posts as supportive.[44] Moreover, by design social media is more accessible than traditional venues for social support, which often involve temporal and even financial investments. For marginalized patients, having the ability to access support without barriers such as transportation and associated costs is significant. Being able to readily access and contribute to conversations surrounding cancer, facilitated by hashtags, on social media can potentially minimize feelings of isolation and engender feelings of community.

However, as described in previous work,[27] we also found that most cancer-related social media posts focus on cancers that predominantly affect women, suggesting that social media does not offer the same appeal as a venue for men’s illness narratives. Conceptualized as a space for social support, this observation follows the general trend that men lack the robust social networks often found among women, both virtually and in real life.[45–47] Given the public nature of social media, or at least the posts we analyzed, it may not be surprising that men are less likely to engage in sharing illness narratives given the norms surrounding masculinity that deter disclosure, even in private.

Our study contributes to this growing body of work through a concurrent analysis of Twitter and Instagram posts. Going beyond previous studies on how Twitter functions as a site for information sharing by individuals or organizations,[20,24,26,29,30] our study emphasizes the role social media plays as a venue for sharing illness narratives. Analyzing both Twitter and Instagram in conjunction allowed us to observe the apparent difference in the tone and thematic content between Instagram and Twitter posts. Specifically, Instagram takes on a narrative form that represents journeys through cancer treatment and subsequent survivorship, whereas Twitter is more factual, leaning towards advocacy, awareness and fundraising. When we consider the form and function of these social media platforms, these observations are not surprising. By design, Instagram solicits images and thereby more complex representations whereas Twitter as a microblogging platform solicits primarily text.

As a whole, we observed highly complex and curated posts, which we suspect may project an unrealistic representation of cancer treatment and survivorship. We hypothesize that these misrepresentations may influence the public’s perception of cancer in ways that are not beneficial to cancer patients or their loved ones. Specifically, such representations may set unrealistic expectations for those beginning treatment and be ultimately a detrimental to psychosocial health when treatment trajectories do not align with these dominant narratives. For those who have completed treatment and have not experienced a return to pre-cancer functional status or appearance, such posts may be alienating and deter individuals from engaging with online communities for cancer patients and survivors, which have been shown to promote quality of life.[14,21] Conversely, posts contrast scarring and hair loss, which are conventionally depicted as tragic, with smiles and hopeful expressions that call into question the experience of cancer treatment as unremittingly negative. The impact of cancer treatment on the body (e.g. removal of nipples) bypasses censorship of nudity on these platforms and allows individuals to share publically both the corporeal and metaphysical transformations brought about through cancer while challenging conventional notions of beauty and censorship. The way individuals who identify as cancer survivors on social media boldly display physical transformations due to cancer treatment may mitigate the trauma associated with scarring, hair loss and the like. The ways in which these posts suggest posttraumatic growth leads us to posit that the sharing of these images can be cathartic. Moreover, social media research on “body positivity”, or the promotion of the appreciation for a wide range of body types and appearances, suggests that posts that challenge socially dominant representations of beauty have positive psychosocial effects on consumers of these images.[48]

As mentioned in previous literature,[49] aligned with this reframing is the undercurrent the term “cancer survivor” may not be representative of the diverse experience of all those who complete cancer treatment. However, posts that explicitly pushed back against conventional notions of health and beauty were not nearly as prominent, let alone popular, as those that focus on a return to a pre-cancer state. In other words, the most salient representations defined survivorship aesthetically (i.e. reconstruction, hair growth, weight gain) as opposed to biomedically (i.e. remission, tumor size, treatment completion). For the sake of privacy, we are unable to share these images. However, posts informing this classification often portrayed dramatic before-and-after images contrasting the aesthetics of treatment and post-treatment.

This study has limitations. While we emphasize survivorship, due to the methodology used here our precision is limited. Although the hashtags used for sampling refer to survivorship, not all do, and we may be capturing posts covering the continuum from diagnosis to treatment to survivorship. Additionally, it is unclear if the prevalence of “celebration” would be the same if we sampled at a time that did not coincide with NCSD. While we chose that day purposively to get to representations of survivorship specifically, future research into cancer and social media would benefit from an exploratory approach to discover the myriad of ways patients represent their experience, from patient to survivor.

Despite the widespread use of social media, social media posts are not representative of the population more broadly. Rather, our findings should be interpreted as capturing the representations and narratives of only those self-selecting individuals who identify as cancer survivors through the content of their social media posts around National Cancer Survivors Day. Specifically, given that users of Twitter and Instagram are younger than the majority of individuals diagnosed with cancer in the United States, we are likely only getting at a subset of cancer survivors. However, despite this limitation, this approach has value. For example, it may help researchers identify personality traits shared among this sub-group that make social media an appealing venue for disclosure and social support, and furthermore trace these to clinical outcomes thus putting it into conversation with studies looking at the impact of openness and resilience on survivorship.

Moreover, while social media is not mediated in the same was as traditional sites or activities of research (e.g. focus groups, support group), we cannot rule out response bias derivative from demand characteristics.[28] Finally, in our initial sampling strategy we did not exclude posts from organizations as we were interested in exploring the conversations surrounding National Cancer Survivors Day broadly. However, we recognize that due to social media algorithms posts from verified, organizational accounts may gain more traction than posts from individuals, potentially skewing our sample. Moreover, these organizational posts may be prescriptive of attitudes that may not necessarily align with those individuals or groups who identify as cancer survivors.

Despite these limitations, we posit that social media can be window into the lives of those individuals who identify as cancer survivors and use social media to represent their experience. This can help researchers and clinicians learn more about the aspirations, needs and general experience with this understudied population that can inform interventions aim at this subpopulation. Furthermore, given the presence of this population on platforms such as Twitter and Instagram, social media may provide a new venue for interventions for this population.

## Conclusion

It is well documented that social media serves as a site for identity-formation and performance,[18,19] wherein individuals both curate their identities as well as pilot narratives and perspectives that may or may not become incorporated into the individual’s conception of self, depending on how well it resonates with society at large. In other words, social media posts represent individuals both as they are and as they are becoming.

Our findings suggest that survivorship is defined fluidly, incorporating elements of physical, emotional and psychological health as well as autonomy. However, despite this variety, the identities of cancer survivors are commonly defined, at least in part, in terms of aesthetics. While undergoing cancer treatment is an unequivocally transformative experience, aesthetic definitions of survivorship close off the multitudinous ways this transformation manifests, potentially alienating and misleading cancer patients who do not experience a return to pre-cancer functional status or appearance. To use our own terms, cancer patients and the public could be misled to believe that cancer treatment always culminates in a rebirth or renewal as opposed to transformation more broadly. Therefore, we need to be cognizant of the norms being set about cancer survivorship on social media, which in its curatorial capacity holds the potential to distort reality. Further research needs to be conducted to assess the validity of this hypothesis and explore more deeply the various ways patients represent and conceptualize cancer survivorship and its impact on the psychosocial health of this growing population.

## Supporting information

S1 Appendix Codebook, definitions and examples. (PDF)

S2 Appendix Image content types and definitions. (PDF)
