# Supplementary material for: Content shared on social media for national cancer survivors day 2018
Source: PLoS One. 2020 Jan 15;15(1):e0226194. doi: 10.1371/journal.pone.0226194 (PMC6961846; doi:10.1371/journal.pone.0226194)
Supplement: S1 Appendix — (PDF) [file pone.0226194.s001.pdf]

Appendix A: Codebook, definitions and examples

Code	Definition
Advocacy	Posts referencing and advertising NCSD, cancer, cancer research, fundraising. The purpose of these posts were to spread general awareness about cancer treatment and prevention, whereas the category “information/education” describes posts with specific information and knowledge regarding these.
Affirmation Statement	Positive text statements
Before/After	Picture before/during treatment and after
Body acceptance	Posts reconciling with body changes due to cancer treatment
Breast cancer	Breast cancer survivors
Casual	Casual posting, day to day life
Childhood cancer	Post showing kids with cancer
Commercial	Advertisements, promotions, etc
Emotion	Post shows strong emotions (e.g., distress, tears)
End of Treatment	Pictures or texts signifying the end of treatment. Usually not a before/after or glamor as the focus is on the end of treatment and what that looks like.
Event	Post documenting or commemorating an event
Family	Pictures of family
Fighter/warrior	post describes cancer as battle and/or survivors as fighters or warriors
Fundraising	Request for donations
Glamor Shot	Pictures looking their best despite the negative effects cancer and treatment.
Gratitude	Post mentions gratitude explicitly
Hair loss	Post shows no hair on head
Heroism	caregivers or survivors described as heroes
Honoring/Remembrance	Post making a tribute to cancer survivors
Information/Education	A post with specific information, news, or other educational material about cancer treatment, prevention and survivorship. Unlike the advocacy category, the information provided here is more specific.
Illness narrative	Story about journey with cancer
Inspiration	Post mentions inspiration explicitly
Identity statement	Post containing explicit statement in text or hashtag about personal identity (e.g., This is me)
Life advice	Post containing advice for life (e.g.,"Find joy in the small things")

Appendix A: Codebook, definitions and examples

Life lessons	Post mentions what cancer taught them about life
Male	Male poster
Nudity	Post showing partial nudity
Organization	A post by an organization on their social media account.
Physical Health	Out in nature or exercise, illustrating their physical health.
Positivity/Hope	Post mentioning positivity explicitly
Religion/faith	Post mentions prayers/ faith/ God/ religion
Repost	A post that has been reposted from another user
Request to be shared	Post that explicitly requests to be shared (e.g., "Share this story by tag your friend/family/lovers")
Scar	Scars or covering up scars.
Strength	Emphasis on strength of survivors
Transformation	Post describing **change** in personality/being as a result of cancer survivorship
Treatment	Picture during treatment (may combine with end of tx to just get all images during or right after tx)
