# Supplementary material for: Content shared on social media for national cancer survivors day 2018
Source: PLoS One. 2020 Jan 15;15(1):e0226194. doi: 10.1371/journal.pone.0226194 (PMC6961846; doi:10.1371/journal.pone.0226194)
Supplement: S2 Appendix — (PDF) [file pone.0226194.s002.pdf]

Appendix B: Image Content Types and Definitions

**Table Image Content Category**	**Definition**
Selfie	Cancer patient posting a photo of themselves
Patient	Family member/friend/loved posting photo of cancer patient
Support Group	Image of cancer support group
Promotion/Advertising	Post aiming to sell something
Family/Friends	Post containing family and friends of cancer patient
Nature	Image of nature
Nutrition	Image of food promoting healthy diet
Doctor	Photo of patient's doctor
Fundraising	Photo or promotional poster to raise money
Fitness	Image of patient exercising or exercise equipment
Information	Photo or poster conveying information about cancer
Other	Image that could not be categorized

Note: Themes are listed by prevalence
